# The Synapse Diversity Dilemma: Molecular Heterogeneity Confounds Studies of Synapse Function

**DOI:** 10.3389/fnsyn.2020.590403

**Published:** 2020-10-02

**Authors:** Seth G. N. Grant, Erik Fransén

**Affiliations:** ^1^Genes to Cognition Programme, Centre for Clinical Brain Sciences, University of Edinburgh, Edinburgh, United Kingdom; ^2^Simons Initiative for the Developing Brain, Centre for Discovery Brain Sciences, University of Edinburgh, Edinburgh, United Kingdom; ^3^Department of Computational Science and Technology, School of Electrical Engineering and Computer Science, KTH Royal Institute of Technology, Stockholm, Sweden; ^4^Science for Life Laboratory, KTH Royal Institute of Technology, Solna, Sweden

**Keywords:** synaptome, LTP, electrophysiology, synapse proteome, synapse heterogeneity, synaptic computation

## Abstract

Recent studies have shown an unexpectedly high degree of synapse diversity arising from molecular and morphological differences among individual synapses. Diverse synapse types are spatially distributed within individual dendrites, between different neurons, and across and between brain regions, producing the synaptome architecture of the brain. The spatial organization of synapse heterogeneity is important because the physiological activation of heterogeneous excitatory synapses produces a non-uniform spatial output of synaptic potentials, which confounds the interpretation of measurements obtained from population-averaging electrodes, optrodes and biochemical methods that lack single-synapse resolution. Population-averaging measurements cannot distinguish between changes in the composition of populations of synapses and changing synaptic physiology. Here we consider the implications of synapse diversity and its organization into synaptome architecture for studies of synapse physiology, plasticity, development and behavior, and for the interpretation of phenotypes arising from pharmacological and genetic perturbations. We conclude that prevailing models based on population-averaging measurements need reconsideration and that single-synapse resolution physiological recording methods are required to confirm or refute the major synaptic models of behavior.

## Introduction

Synapse diversity has been known for many decades from pharmacological, physiological and neurochemical studies that have led to the standard classifications of excitatory and inhibitory synapses and different neurotransmitter systems. In the past two decades, studies of the synapse proteome have revealed a high degree of molecular complexity (Husi et al., 2000; Collins et al., 2006; Coba et al., 2009; Bayes et al., 2011, 2012, 2017; Distler et al., 2014). A typical synapse in the mammalian brain occupies a volume of 1 μm^3^ (Bayes et al., 2011) and can potentially house several million individual protein molecules. Approximately 10% of the proteins encoded by the ∼23,000 genes in the human genome are found in synapses.

Proteins are not expressed equally in all synapses; instead, different synapses (types and subtypes) express combinations of proteins (Husi et al., 2000; Micheva et al., 2010; Frank et al., 2016, 2017; Zhu et al., 2018; Cizeron et al., 2020). There is a potentially vast synapse diversity that could arise from the combinatorial expression of synapse proteins (Grant, 2007, 2018a,b; Micheva et al., 2010; O’Rourke et al., 2012; Zhu et al., 2018; Cizeron et al., 2020). Molecularly distinct synapses are differentially distributed within the dendritic tree of individual neurons, and different neurons (even within the same class) have different synapse distributions. Every brain region is characterized by a “signature” of synapse composition (Zhu et al., 2018; Cizeron et al., 2020), which together result in a 3D spatial architecture of the brain, known as the synaptome architecture (Zhu et al., 2018; Cizeron et al., 2020). We recently studied the brain-wide distribution of excitatory synapse types across the mouse lifespan and observed temporal trajectories in synapse parameters and regional compositional signatures (Cizeron et al., 2020). Synapse diversity and its organization into the spatiotemporal lifespan synaptome architecture (Cizeron et al., 2020) most likely reflect a concerted set of genetic programs (Frank et al., 2016, 2017; Frank and Grant, 2017; Skene et al., 2017; Zhu et al., 2018; Grant, 2019; Cizeron et al., 2020).

**FIGURE 1:**
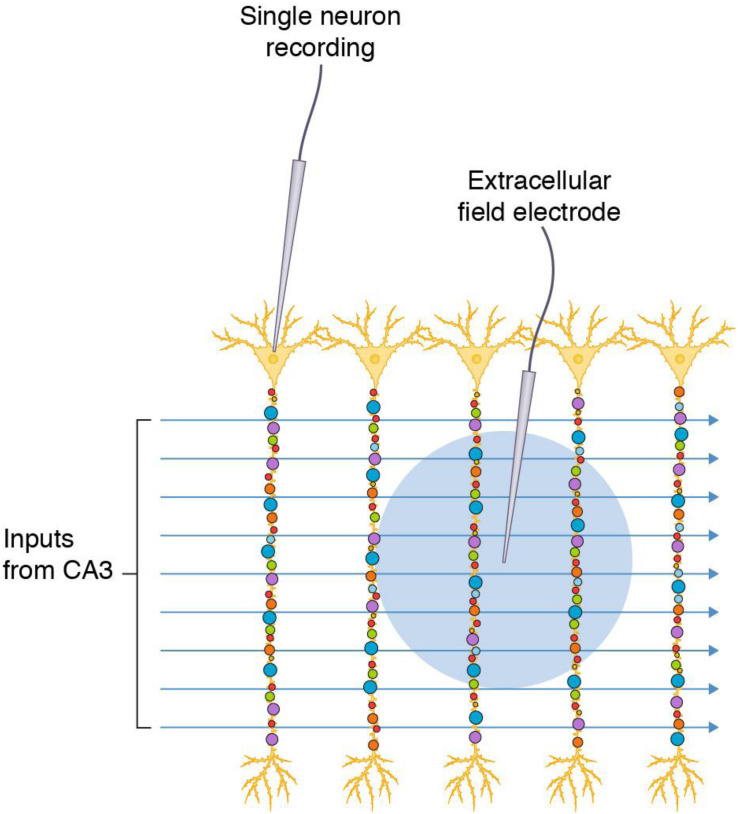
Commonly used electrophysiological methods record responses from synapse populations and do not inform on individual synapses. Populations of diverse synapses in the CA1 stratum radiatum of the hippocampal formation (small colored circles) are distributed on the apical dendrites of pyramidal neurons. Axon inputs from CA3 are stimulated and recording electrodes access populations of synapses in a single neuron by direct recording or populations of neurons by recording in the extracellular space (blue circle indicates the area from which synaptic potentials are detected).

The hippocampal formation of the mammalian brain has attracted much attention as an experimental preparation because its circuitry is amenable to electrophysiological recording of synaptic transmission (Bliss and Collingridge, 1993; Basu and Siegelbaum, 2015; Nicoll, 2017). Slices of hippocampal tissue can be maintained in an organ bath and the strength of synaptic transmission is measured using a stimulating electrode placed into the extracellular space of an afferent fiber bundle (e.g., Schaffer collateral-commissural pathway that projects from the CA3 region to CA1 stratum radiatum) and a recording electrode placed in the dendritic population of apical dendrites of the postsynaptic pyramidal neurons (e.g., CA1 stratum radiatum, CA1sr). Stimulation protocols typically trigger action potentials in many axon fibers that travel to the presynaptic terminal and cause release of neurotransmitter (glutamate) onto dozens to hundreds of postsynaptic dendritic spines (Figure 1). The electrode records the sum of the individual postsynaptic responses (field excitatory postsynaptic response, fEPSP), which is used as the measure of synaptic strength. Other methods record from the soma or dendrites of individual neurons (e.g., cell-attached patch electrodes) and sum the synaptic responses (Figure 1).

Synapse diversity within hippocampal CA1 pyramidal neurons has been described using molecular and morphological approaches. Dye filling experiments show that a single pyramidal neuron in the rat contains ∼32,000 synapses, of which >90% are excitatory (Megias et al., 2001). Molecular studies of these excitatory synapses using synaptome mapping approaches, which quantify the intensity, size and shape of synapses expressing postsynaptic scaffold proteins PSD95 and SAP102, show that the CA1sr has the highest synaptic diversity of any mouse brain region (Zhu et al., 2018; Cizeron et al., 2020). Postsynaptic proteins are differentially distributed along the length of the apical dendritic arborization of CA1 pyramidal neurons, producing synapses of different sizes and amounts of protein organized into gradients (Broadhead et al., 2016; Zhu et al., 2018; Cizeron et al., 2020). Comparison of the dendritic arborizations of adjacent neurons along the medial-to-lateral axis also shows a gradient of synaptic parameters (Zhu et al., 2018; Cizeron et al., 2020). Gradients of diverse synapses are also observed in the striatum and regions of the neocortex (Zhu et al., 2018). Thus, electrophysiological recordings measure the population average of these heterogeneous synapses. The recordings not only average the responses and lose the specific information from individual synapses, but also fail to inform on the spatial location of the different synapses.

The problem of population-averaged assays in biological systems has long been recognized in the context of cellular diversity (Altschuler and Wu, 2010). The overriding assumption is that the population average represents the mechanism(s) operating within individual cells (or synapses) within the population. Population-averaged measurements are known to confound the interpretation of cellular signaling pathways and the roles of cellular subtypes in physiological processes and to poorly reflect the internal states of the majority of the cells, any subpopulation of cells, or even any single cell (Altschuler and Wu, 2010). These problems equally apply to the biology of synapse heterogeneity but have received little attention (O’Rourke et al., 2012).

Population-averaged recordings of synapse physiology that are correlated with behavioral measures assume that all synapses have equal relevance to the behavioral output. However, it is possible, perhaps even highly likely, that only some synapses are relevant for a particular behavior (referred to below as “behavior-relevant synapses”). Similarly, studies of cellular heterogeneity have demonstrated the important roles of small subpopulations of cells or single cells with the population (e.g., cancer cells) (Altschuler and Wu, 2010). The spatial organization of synaptic heterogeneity is important because the physiological activation of heterogeneous excitatory synapses produces a non-uniform spatial output of synaptic potentials (Grant, 2018b; Zhu et al., 2018; Cizeron et al., 2020), and this introduces a host of problems for the interpretation of measurements obtained from a population-averaging electrode. With this background, we will consider how synapse diversity and its spatial organization impact on the interpretation of physiological, genetic, pharmacological and behavioral studies.

## Functional Considerations of Heterogenous Excitatory Synapses

**FIGURE 2:**
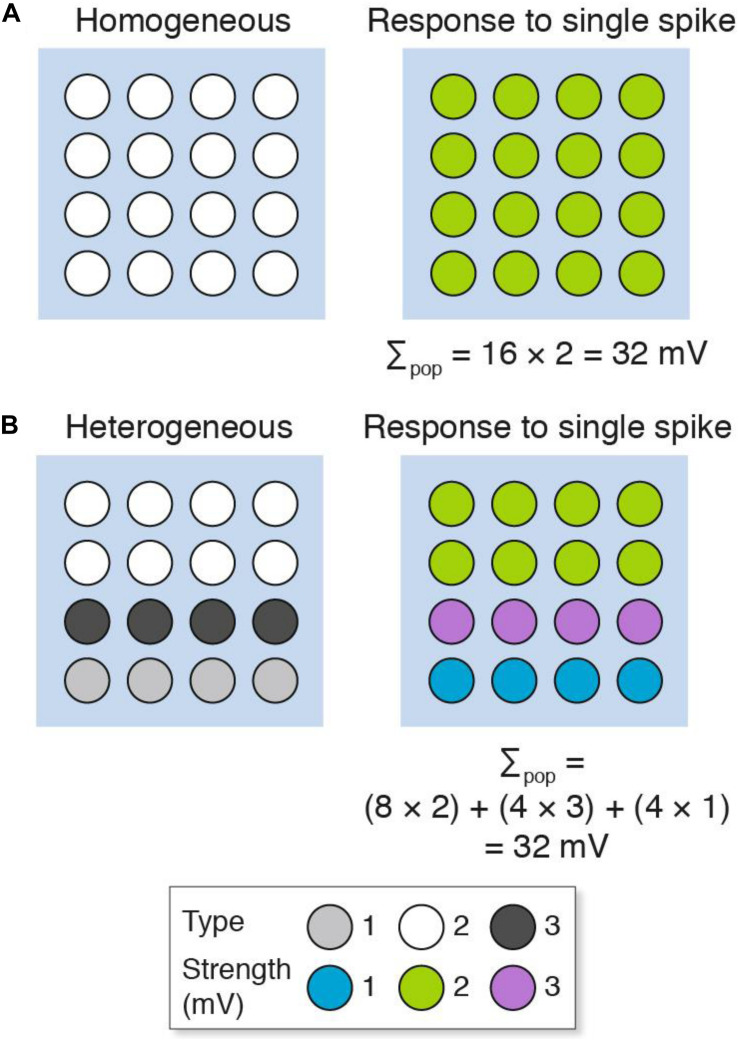
Populations of **(A)** homogeneous and **(B)** heterogeneous synapses can show the same overall response. The key shows three synapse types and their strength. Σ_pop_, summed response for each population.

We will first consider a simple model that contrasts a population of homogeneous with heterogeneous excitatory synapses recorded using a single electrode (Figure 2). The populations consist of 16 synapses, each represented as a circle. The “homogeneous population” is composed of one type of synapse (Figure 2A), whereas the “heterogeneous population” comprises three types (Figure 2B). These synapse types have different molecular compositions and physiological properties: a single action potential generates a 1 mV potential from type 1 (a weak synapse), 2 mV from type 2 (a medium strength synapse) and 3 mV from type 3 (a strong synapse). There is a non-random spatial organization (synaptome architecture) to the heterogeneous population, where the top two layers are type 2 synapses, the third layer is type 3 and the fourth layer is type 1 (Figure 2B). When these two populations are stimulated with a single action potential, there are distinct spatial maps of synaptic responses observable at single-synapse resolution. However, the population recording does not detect the spatial differences (or synapse diversity) and the summed response (Σ_pop_) in both populations is the same (Σ_pop_ = 32 mV). In other words, electrophysiological methods that record population measurements are blinded to the diversity of synapses and their spatial organization.

**FIGURE 3:**
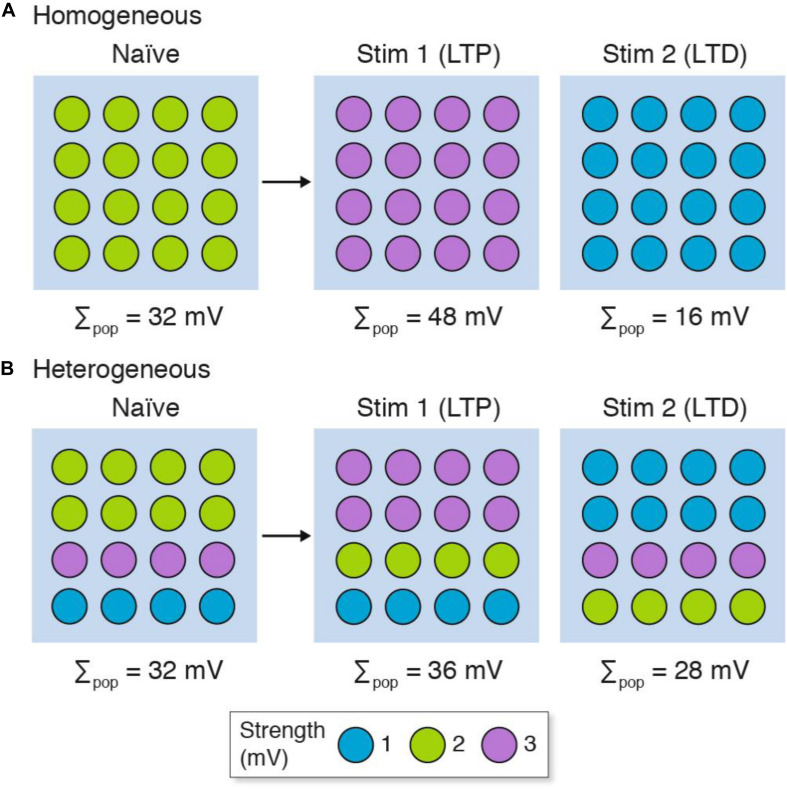
LTP and LTD in populations of **(A)** homogeneous and **(B)** heterogeneous synapses. The strength of the synapse populations in Figure 2 are shown before (naïve) and after LTP (Stim 1) and LTD (Stim 2) induction. Although both homogeneous and heterogeneous populations show LTP and LTD, only some synapses in the heterogeneous populations reflect the population measure and subpopulations of synapses can show opposite phenotypes (i.e., LTD in individual synapses when the population shows LTP, and vice versa). The key shows three synapse types and their strength. Σ_pop_, summed response for each population.

Next we will consider how population recordings could confuse the interpretation of synaptic plasticity in heterogeneous populations of synapses (Figure 3). Here we will contrast two stimulation protocols: one that results in long-term potentiation (LTP) and another that results in long-term depression (LTD). Both the homogeneous and heterogeneous populations show LTP (Σ_pop_ increases from control) and LTD (Σ_pop_ decreases from control). However, unlike the homogeneous population where all the synapses either strengthen or weaken, the heterogeneous populations show physiological diversity at the single-synapse level: some synapses strengthen and others weaken. If the specific synapses (or subsets) within each population were to have distinct physiological outputs (for example, because of their location within the dendritic tree) then the population recording could not be relied upon to inform us if their output was increased or decreased in either the LTP or LTD experiment.

The failure of population recordings to discriminate spatial effects of heterogeneous synapse populations has important implications for those experiments that attempt to correlate physiological and behavioral properties. For the purposes of this discussion, we will assume that a subset of synapses within the population drive a circuit that controls the behavior (behavior-relevant synapses). An increase in synaptic strength recorded in the whole population of synapses (LTP) would not necessarily correlate with an increase in synaptic strength in the behavior-relevant synapses and so there would not be a correlation between LTP and learning. Thus, if we assume that LTP is the causal mechanism of learning (Bliss and Collingridge, 1993), then synapse diversity could be invoked to explain why dissociations between the direction or strength of change in LTP and learning do not necessarily refute that model. However, the LTP-learning model is itself based on population-averaging measurements (Bliss and Lømo, 1973; Bliss and Collingridge, 1993; Basu and Siegelbaum, 2015; Nicoll, 2017) and must therefore be questioned in the same light. If we do not start with the *a priori* assumption that LTP is the mechanism of learning, then population-averaging recordings of synapses cannot be used to support the model because it cannot be safely assumed that all synapses are functioning in the same way, as shown in Figure 3B. Therefore, experiments that do not resolve the physiological properties of individual synapses and rely on population measurements cannot be used to support or rebut the LTP model of learning.

A substantial body of literature describes changes in LTP and LTD during the postnatal developmental period and has suggested that these changes are important for learning in critical periods (Fox, 1992; Feldman et al., 1999; Kirkwood et al., 1995; Ge et al., 2007). Our recent study of the lifespan synaptome architecture shows a dramatic increase in excitatory synapse diversity during the postnatal developmental period, with every brain region undergoing major compositional changes in heterogeneous synapses (Cizeron et al., 2020). Thus, although the electrophysiological studies have been interpreted as changes in long-term synaptic strength through synaptic plasticity of existing synapses, a radical suggestion, which remains formally possible, is that there could be circumstances where there are no changes in long-term synapse strength mediated by plasticity but changes in the composition of synapse populations. Nevertheless, stimulation of single presynaptic terminals can induce short-term plasticity indicating that at least this form of plasticity occurs at single synapses (Vandael et al., 2020). Untangling the relative contribution of synaptic plasticity and changing populations of synapses to the long-term changes in synaptic strength will require physiological and molecular studies of synapses at single-synapse resolution.

## Interpretation of Pharmacological Perturbations of Diverse Synapses

**FIGURE 4:**
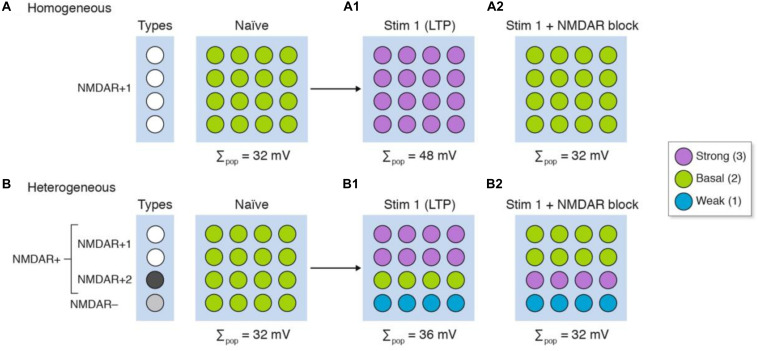
The differing molecular composition of individual synapses confounds the interpretation of signaling mechanisms. Three types of synapse are illustrated in four rows: NMDAR+ synapses, which express the NMDA receptor, are divided into NMDAR+1 synapses that have an enzyme used for NMDAR induction of LTP, and NMDAR+2 synapses which lack the enzyme; and NMDAR− synapses that do not express NMDA receptors but can be weakened by synaptic stimulation. After stimulation (Stim 1), LTP is induced in all the homogeneous synapses **(A1)** but in only half the heterogeneous synapse populations **(B1)** even though LTP is measured in the whole population. The same experiment performed in the presence of an NMDA receptor blocker **(A2,B2)** shows that the population measure of LTP is blocked. However, there are synaptic physiological changes in the heterogeneous population that remain undetected **(B2)**.

It has long been known that drugs bind to proteins, and because synapses contain different proteins then drugs will target different synapses. This logic has been at the heart of the majority of neuropharmacological therapies and interventions that target neurotransmitter systems. Here we will illustrate how synapse diversity can confound the interpretation of pharmacological experiments. We will use the LTP models in Figure 3 and incorporate the NMDA receptor (NMDAR) into our homogeneous and heterogeneous synapses. For simplicity, we will assume the standard position in the literature – that patterns of neural activity can activate the NMDAR and postsynaptic signaling pathways lead to strengthening of synapses (Nicoll, 2017). In our model of homogeneous synapses (Figure 4A), which all express the NMDAR (NMDAR+1), LTP is induced in all synapses (Figure 4A1) and pharmacological blockade of NMDAR prevents LTP induction (Figure 4A2). In our model of heterogeneous synapses (Figure 4B), we have synapses that express (NMDAR+) and those lacking (NMDAR−) NMDARs (Figure 4B). The NMDAR+ synapses are divided into two groups: NMDAR+1 synapses, which are the same as those in the homogenous population model (Figure 4A); and NMDAR+2 synapses, which fail to produce LTP when stimulated. Examples of subtypes of NMDA receptors with differential protein interactions and roles in LTP have been described (Ryan et al., 2013; Frank et al., 2016, 2017; Frank and Grant, 2017). As shown in Figure 4B1, stimulating this heterogeneous population of synapses produces LTP in the overall population, whereas at the single-synapse level we see LTP in only half the synapses (NMDAR+1), with the other synapses either unchanged (NMDAR+2) or weakened (NMDAR−).

Next we will repeat this experiment in the presence of a drug that blocks the NMDAR (Figures 4A2,B2). Both homogeneous and heterogeneous populations show no overall change in synaptic strength; in other words, LTP is blocked. However, on closer inspection of individual synapses, we find that within the heterogeneous population some synapses show LTP (NMDAR+2) whereas others show LTD (NMDAR−). These simulations show that in an experiment in which there is diversity in the expression of the NMDAR and downstream signaling molecules, a misleading interpretation can be drawn of the synaptic changes within the population. As noted above, if these subpopulations are of differing relevance to behavioral outputs then the population measures would give a misleading interpretation of both the electrophysiological and pharmacological data. The scenarios we portray are not unrealistic as studies of spike timing-dependent plasticity show that a given pattern of pre- and post-synaptic activation induces LTP at some synapses and induces LTD at other synapses (Brzosko et al., 2019). Moreover, the neuromodulatory neurotransmitters can convert the LTD to LTP and vice versa (Brzosko et al., 2019).

In addition to considering the action of drugs that target neurotransmitters these principles apply to signaling and metabolic enzymes, which are known to have differential distributions (Roy et al., 2018a,b). If synapses showed different rates of protein synthesis then protein synthesis inhibitors would exert different phenotypes on individual synapses. There are well-documented examples of dissociations between protein synthesis and long-term memory, which remain unexplained (Routtenberg and Rekart, 2005; Gold, 2008), potentially because of synapse diversity.

## Interpreting Genetic Perturbations in Diverse Synapses

**FIGURE 5:**
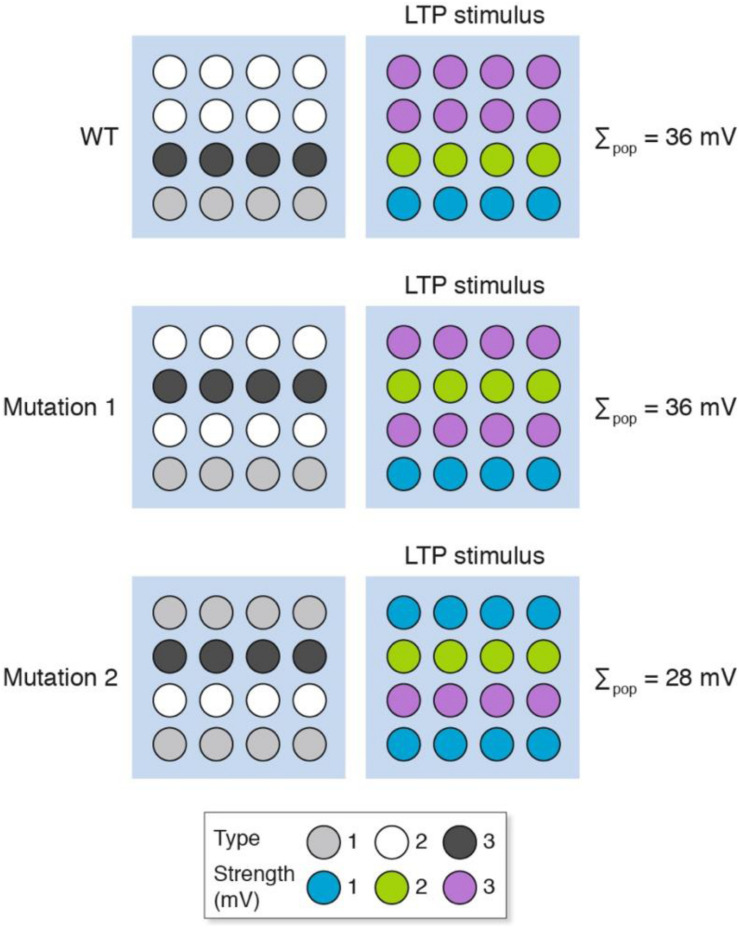
Synaptome reprograming by gene mutations alters the spatial distribution of heterogeneous synapses. The wild-type synaptome map, which is composed of three synapse types, provides an overall population measure (Σ_pop_) after LTP induction of 36 mV. With mutation 1, the spatial distribution of synapses has changed but the total LTP is the same as for wild type (Σ_pop_ = 36 mV). Mutation 2, however, has altered the representation of synapse types (more type 1 and fewer type 2) and an LTP-inducing stimulus shows less LTP than the wild type.

Genetic perturbations (e.g., knockout mice, knockdown approaches) will change the molecular composition of individual synapses and thereby alter their signaling properties in a way that is similar to the pharmacological model presented above (Figure 4). In addition to this mechanism, we have previously described “synaptome reprograming” in mice carrying gene mutations (Zhu et al., 2018; Grant, 2019). Broadly speaking, synaptome reprograming changes one heterogeneous population of synapses into a different population (Figure 5). We will consider two versions of synaptic reprograming that occur with gene mutations: (i) where the spatial location of the different synapses has changed (Figure 5, mutation 1); and (ii) where the numbers of synapse types and their spatial location have changed (Figure 5, mutation 2).

After an LTP-inducing stimulation, the response of mutation 1 is the same as wild type, but the mutant synaptome produces different outputs from those synapses located in rows 2 and 4. If these were behavior-relevant synapses, then this would produce behavioral differences to the wild type without an apparent difference in LTP. The response of mutation 2 is a different spatial output to the wild type and mutation 1 and an overall lower LTP. The behavioral output will depend on the relative importance of synapses in different locations. Together, these examples illustrate how genetic mutations could produce a change in LTP and have any number of effects (increase, decrease or no change) on behavior.

These simulations have implications for the interpretation of population recording in mutant organisms. In the presence of synaptic diversity it is not possible to conclude the physiological properties of individual synapses or the potential contribution to a behavioral output without knowing the physiological changes in each synapse and the spatial location of the synapse. With synaptome reprograming, the spatial reorganization of synapse types complicates the interpretation of basal physiological differences between wild type and mutant, and of differences that arise after a stimulation protocol that induces LTP.

## Discussion

The vast majority of electrophysiological studies of synaptic function employ methods that record from populations of synapses. Moreover, EEG and fMRI signals are assumed to stem largely from populations of synapses. Data from these experiments have been highly influential; for example, they have been used to support the hypothesis that an increase in stable synaptic strength can account for learning and for how behaviors change during development. These models are based on the assumption that the synapses are homogeneous and therefore the population measure can be extrapolated to reflect the physiology of the individual synapses. However, in the presence of synapse diversity, the population measure does not accurately inform on the physiological properties of individual synapses. As we have argued, there can be a dissociation between the population measure and the changes in subsets of synapses. Moreover, in the presence of a genetic, pharmacological or other biochemical perturbation that changes the molecular composition of synapses and/or their spatial location, the interpretation of the data is further confounded.

Synapse diversity interferes in many different ways with the interpretation of electrophysiological experiments that record populations of synapses. Phenotypic dissociations between electrophysiology and behavior using pharmacological and genetic approaches are confounded. Indeed, there have been many examples where LTP and learning have been dissociated with pharmacological and genetic approaches. These dissociations could be explained by synapse diversity and synaptome reprograming. Although synapse diversity and synaptome reprograming could be used to dismiss a dissociation, we need to recognize that the null hypothesis itself (that LTP is the causal mechanism of learning) is also subject to the same confound because the experiments that have shown the correlation between synapse strength and learning are themselves based on population measurements of synapse physiology.

An important area of molecular neuroscience has been the dissection of signaling pathways from neurotransmitter receptors. These experiments have almost universally used synapse population measures (e.g., hippocampus slices bathed in drugs), and the phenotypes of different molecular perturbations are assumed to reflect the “pathways” inside the homogenous synapses. However, the molecular targets of these drugs and the proteins comprising the signaling pathways can differ between synapses, and the physiological outputs could therefore represent changes in the relative contributions of different synapse types rather than the efficacy of the putative pathway. This problem has previously been described in the context of cellular heterogeneity (Altschuler and Wu, 2010). Synapse electrophysiology is not the only area of synaptic biology that is bedeviled by synapse diversity. Many biochemical studies involve extracting proteins from populations of synapses (e.g., western blotting) and neurochemical approaches obtain measurements from large populations of synapses.

It is also important to recognize that homogenous synapses can have differential physiological outputs depending on their spatial location in the dendritic tree (Spruston, 2008). When considering the physiological importance of synapses, it is therefore necessary to consider the spatial location and the molecular, morphological and functional characteristics of each synapse. This underlines the conceptual and practical importance of the synaptome architecture (Grant, 2018b; Zhu et al., 2018; Cizeron et al., 2020). The first synaptome maps that describe the synaptome architecture of the mouse and human brain are now emerging (Zhu et al., 2018; Cizeron et al., 2020; Curran et al., 2020). In the same way that neuronal types are being reclassified and atlased using genomic methods, we expect that there will be new classifications of synapses and synaptome atlases, which will be key reference resources. Linking synaptome and connectome atlases will enable an understanding of the physiological properties of specific circuits and synapse types to be integrated with electrophysiological and behavioral mechanisms.

Until single-synapse resolution physiological responses can be correlated with the known molecular constituents of the recorded synapses, it will not be possible to safely interpret population-based physiological studies. Importantly, the current models of synaptic mechanisms and their relevance to behavior will need to be re-examined before we can have confidence in their validity. This does not mean we are arguing against populations of synapses as important carriers of information, but rather to emphasize the need for identification of synapse type. In this regard, optical approaches to molecular imaging offer an increasingly powerful set of tools capable of resolving the molecular composition of individual synapses and their functional properties. Synaptic proteins could be genetically labeled (using fluorescent proteins (Zhu et al., 2018) or self-labeling tags (Masch et al., 2018)) to identify synapse subtypes together with simultaneous optical recording using genetically encoded functional reporters (e.g., Ca^2+^, voltage indicators), or dyes that fill dendritic spines, or labels that reveal the dynamic nanoarchitecture of synapses (Masch et al., 2018; Wegner et al., 2018). Electrophysiological stimulation of individual presynaptic terminals of mossy fibers paired with postsynaptic recording in the CA3 region using patch electrodes is a powerful approach that can be coupled with Ca^2+^ chelators that modify the biochemical properties of individual synapses (Vyleta and Jonas, 2014) and has the potential to be combined with molecular labeling methods that distinguish individual synapses. Ideally, the recording systems would not use “whole cell recording” of individual postsynaptic events, but direct recording from individual postsynaptic terminals. Although the characterization of synapse diversity and synaptome architecture raises problems for the existing literature, it also opens completely new models of physiology and behavior based on the functional diversity of molecularly distinct synapses (Grant, 2018b; Zhu et al., 2018).

## Data Availability Statement

All datasets generated for this study are included in the article, further inquiries can be directed to the corresponding author/s.

## Author Contributions

All authors listed have made a substantial, direct and intellectual contribution to the work, and approved it for publication.

## Conflict of Interest

The authors declare that the research was conducted in the absence of any commercial or financial relationships that could be construed as a potential conflict of interest.
